# Molecular Insights on New Psychoactive Substances (NPSs)

**DOI:** 10.3390/ijms23063282

**Published:** 2022-03-18

**Authors:** Francesco Paolo Busardò, Simona Pichini

**Affiliations:** 1 Analytical Pharmacotoxicology Unit, National Centre on Addiction and Doping, Istituto Superiore di Sanità V.Le Regina Elena 299, 00161 Rome, Italy; 2 Department of Excellence of Biomedical Sciences and Public Health, “Politecnica delle Marche” University of Ancona, Via Tronto 10/a, 60126 Ancona, Italy; simona.pichini@iss.it

Currently, more than 1000 molecules have been classified as New Psychoactive Substances (NPSs), and it is reported that, every year, this number increases with new classes of compounds and/or newer generations of NPS families [1,2].

To document exposure to NPSs, clinical and forensic toxicologists have attempted to measure both parent compounds and their metabolites. However, due to the fact that new NPSs emerge on illicit markets every year, when they first appear, their pharmacokinetic properties, their toxicity, and their consequent risks to consumer health are unknown [3]. The objective of this Special Issue is to provide the most updated molecular knowledge in the field of NPS.

In this Special Issue, particular attention is paid to studies concerning two classes of NPS that dominated illegal drug trafficking: synthetic cannabinoids and synthetic cathinones.

Even though international laws have banned the latter to limit their spread, the overall availability of new synthetic cathinones has not decreased. Recently, mexedrone (3-methoxy-2-(methylamino)-1-(4-methylphenyl)propan-1-one), α-PVP (alpha-pyrrolidinopentiophenone), and α-PHP (alpha-pyrrolidinohexanophenone), and other cathinones have been associated with fatal and non-fatal intoxications [4], emphasizing the importance of knowing their pharmaco-toxicological profiles. In this regard, Lenzi and coworkers evaluated the mutagenicity of these compounds in an in vitro study on human lymphoblastoid TK6 cells. Mexedrone demonstrated a mutagenic potential different from the other two compounds, in which mutagenicity was shown in their metabolites instead. In this regard, the fact that α-PVP and α-PHP produced metabolites with mutagenic capacities allowed the authors to ascribe the ability to induce chromosomal aberrations, an aspect linked to long-term toxicity, to these two synthetic cathinones, since the close link between mutations and chronic-/neuro-degenerative pathologies is well recognized [5].

In an in vitro study, Kolaczynska et al. investigated the pharmacological profiles of several first- and second-generation pyrovalerone cathinones such as 3,4-methylenedioxpyrovalerone (MDPV), 3,4-methylendioxy-α-pyrrolidinobutiophenone (MDPBP), pyrovalerone, α-pyrrolidinopropiophenone (α-PPP), α-pyrrolidinopentiophenone (α-PVP), α-pyrrolidinohexanophenone (α-PHP), 4-fluoro-α-pyrrolidinobutiophenone (4-fluoro-α-PBP), N-ethylamine-hexanophenone (NEH), 3,4-methylenedioxy-α-pyrrolidinopropiophenone (MDPPP), 4-methoxy-α-pyrrolidinopropiophenone (MOPPP), 3,4-methylenedioxy-α-pyrrolidinohexanophenone (MDPHP), 4-methyl-α-pyrrolidinohexanophenone (MPHP), and 4-methyl-α-pyrrolidinopropiophenone (4-MePPP). All of the compounds were potent inhibitors of norepinephrine and dopamine uptake transporters and, to a far lesser extent, a serotonin uptake transporter [6].

A third in vitro study on pyrovalerone derivatives was carried out by Carlier and coworkers [7]. Human hepatocyte incubations followed by liquid chromatography–high-resolution tandem mass spectrometry and data-mining software were used to determine the metabolite profiles of α-pyrrolidinohexaphenone (α-PHP) and 4′′-fluoro-α-pyrrolidinovalerophenone (4F-α-PVP). α-PHP dihydroxy-pyrrolidinyl, α-PHP hexanol, α-PHP 2′-keto-pyrrolidinyl-hexanol, and α-PHP 2′-keto-pyrrolidinyl were identified as markers of α-PHP use, and 4F-α-PVP dihydroxy-pyrrolidinyl, 4F-α-PVP hexanol, 4F-α-PVP 2′-keto-pyrrolidinyl-hexanol, and 4F-α-PVP 2′-keto-pyrrolidinyl were identified as markers of 4F-α-PVP use.

The last in vitro study, presented by Sogos and colleagues, focused not only on a synthetic cathinone, 40-methyl-alphapyrrolidinoexanophenone (3,4-MDPHP), but also on a phenethylamine, 2-chloro-4,5-methylenedioxymethamphetamine (2-Cl-4,5-MDMA), and on a fentanyl, as a reference compound for synthetic opioids [8]. The in vitro toxic effects of these three compounds were evaluated in dopaminergic-differentiated SH-SY5Y cells, a thrice-subcloned cell line derived from the SK-N-SH neuroblastoma cell line. The experiments performed provided evidence that, whereas 3,4-MDPHP elicited cell death by necrosis, 2-Cl-4,5-MDMA activated apoptotic processes and fentanyl triggered cell death through both mechanisms, confirming the different modes of cell death caused by the different compounds.

As reported above, the second most commonly consumed class of NPSs is synthetic cannabinoids, which similar to that reported for synthetic cathinones, create health concerns due to their severe adverse events and enhanced toxicity [9]. Whereas several analytical methods can detect these compounds in cases of acute and fatal intoxications [10], information about the disposition of synthetic cannabinoids and their metabolites in consumers’ biological matrices is still scarce.

For the first time, La Maida et al. identified and quantified JWH-122, JWH-210, UR-144, and their principal metabolites in the oral fluid of consumers [10]. A combination of an initial last-generation gas chromatography–mass spectrometry (GC-MS) screening method for the determination of the parent compounds in oral fluid and an ultrahigh-performance liquid chromatography–high-resolution mass spectrometry (UHPLC-HRMS) confirmatory method for the quantification of JWH-122, JWH-210, UR-144, and their metabolites was set up and validated for this purpose. After smoking JWH-122 or UR-144, and 3 mg of JWH-210, the maximum concentrations of the parent compounds were measured at 20 min after inhalation. The metabolites, JWH-122 N-(4-OH), JWH-210 N-(4-OH), and JWH-210 N-(5-OH) were quantified in oral fluid by UHPLC-HRMS, while JWH-122 N-(5-OH) and UR144 were only detectable in traces.

Starting from the above-reported analytical method, the same research group proposed a screening method for urinalysis of classical drugs of abuse; NPSs, including new synthetic opioids; and their main metabolites using a fast sample extraction [11]. Additionally, in this case, two different analytical technologies—high-sensitivity GC-MS and UHPLC-HRMS—were used for a screening analysis of classic drugs and new psychoactive substances, and their metabolites in the urine of people with heroin addictions under methadone maintenance therapy. Due to well-stocked and up-to-date libraries of the spectra, a combined method could identify up to around one thousand different compounds, and in urine samples from 296 patients with a history of opioid use disorder, about 80 different psychoactive substances and/or metabolites plus several prescription drugs were identified. An analytical method used to screen for a huge and upcoming number of NPS in urine is strongly needed in suspected NPS-related fatalities or acute intoxication/exposure occurring in emergency departments and drug addiction services [12].

This Special Issue closes with an extensive, updated, and well-articulated review on toxicology, chemical structures, and reported cases of overdose related to psychoactive tryptamines by Malaca et al. [13].

Even if psychoactive tryptamines are among less prevalent NPSs [1,2], there are signs of a recent increase in their overall use, with very scarce information on the newest compounds. The authors showed that, currently, the most prevalent tryptamines are 5-methoxy-N,N-diisopropyltryptamine (5-MeO-DiPT), 5-methoxy-N,N- diallyltryptamine (5-MeO-DALT), and dimethyltryptamine (DMT). Twenty two new analytical methods were detailed for used in identifying/quantifying tryptamines and metabolites in biological samples, and few intoxication cases and even fewer fatalities due to tryptamines consumption were reported in North America and the EU. Nevertheless, it was shown that the morbidity accompanying tryptamine intake is considerable and that it is critical for clinicians and laboratorians to be informed of the latest data on this public health threat.

In conclusion, we hope that this issue will contribute to advancements not only in molecular knowledge but also in the understanding of toxicity, health risks, and analytical challenges in the field of NPS.
